# Medial Tibial Stress Syndrome: A Scoping Review of Epidemiology, Biomechanics, and Risk Factors

**DOI:** 10.7759/cureus.81463

**Published:** 2025-03-30

**Authors:** Mohamed A Saad, Jamal M Jamal, Abdulrazzaq T Aldhafiri, Salem A Alkandari

**Affiliations:** 1 Physical Medicine and Rehabilitation, Physical Medicine and Rehabilitation Hospital, Ministry of Health, Kuwait City, KWT; 2 Physical Medicine and Rehabilitation, Ahmadi Hospital, Zahra, KWT

**Keywords:** exercise-induced leg pain, medial tibial stress syndrome, overuse injury, scoping review, shin splints

## Abstract

Regular physical activity confers numerous health and social benefits; however, activity-related lower-limb overuse injuries are prevalent. Medial tibial stress syndrome (MTSS) is a common overuse injury of the lower extremity. It is frequently observed in athletes and in military personnel. It involves exercise-induced pain over the anterior tibia and is an early stress injury in the continuum of tibial stress fractures (TSF). This scoping review aims to synthesize and characterize the literature on MTSS, identify knowledge gaps, and propose future research directions. We conducted a scoping review of the literature, which was informed by the Joanna Briggs Institute and the Arksey and O’Malley methodology, by searching four databases until December 31, 2023. The references were screened by two authors based on their titles, abstracts, and full texts to ensure that they met the inclusion criteria. Data related to the research objectives were extracted, analyzed, and reported numerically and descriptively. Thirty-seven studies were included in the current review, most of which recruited athletes as the study participants (67.6%). This was followed by articles about military members (16.2%). The highest prevalence of MTSS has been reported among recreational marathon runners in India (69.5%). The highest incidence of MTSS was reported in a German study (35.7%). Two studies reported that participants with MTSS had a higher body mass index (BMI) than those without MTSS, and one study found a significant correlation between BMI and the time required for complete recovery. Two studies reported that individuals with greater experience in athletic activities were at a lower risk of developing MTSS later in their careers. In contrast, another study reported that ultramarathon running increases the incidence of MTSS overuse injuries. Traction theory as a cause of MTSS was examined in five studies, and the mean navicular drop test (NDT) score was assessed in three studies. Biomechanical evaluation of ankle disorders and MTSS was performed in five articles and hip abnormalities in four articles. This review identifies research areas on the influence of epidemiological, risk, and biomechanical factors on MTSS development. Such research could inform counseling for patients concerned about MTSS risk factors and curative measures, as well as optimize surveillance and subsequent management of MTSS outcomes.

## Introduction and background

Definition

Leg is defined as "the inferior part of the lower extremity between the knee and the ankle, and it consists of two bones: the tibia and fibula" [1]. Exercise-induced leg pain (EILP) is a broad term describing leg soreness caused by physical activity [2]. Medial tibial stress syndrome (MTSS) is also known as "shin splints." It is an anterior tibial EILP and an early stress injury in the continuum of tibial stress fractures (TSF). This syndrome frequently occurs in athletes and in military personnel [3].

Historical context

MTSS, initially recognized in athletes in 1913 as "spike soreness," was once considered a type of stress fracture (SF). Over time, it has been identified as a distinct condition with unique clinical features [4]. Devas [5] was one of the first physicians to examine "shin soreness" in athletes, although, like earlier researchers, he considered it to be a type of SF. In 1967, Slocum presented a comprehensive review of the disease, highlighting MTSS as a distinct injury with a distinct clinical picture and pathology [6]. In the 1980s, several nuclear medicine investigations aided in improving the diagnosis of MTSS [4]. The emergence of bone scanning technologies, notably magnetic resonance imaging (MRI), has resulted in the medical community renaming shin splints as MTSS. This modern diagnostic capability has contributed to the development of precise criteria for MTSS [7].

Pathophysiology

However, the pathophysiology of MTSS remains to be elucidated. One hypothesis posits that pain is attributed to periosteal inflammation resulting from excessive traction on either the tibialis posterior or the soleus muscle. This concept is reinforced by the bone scintigraphy results, which reveal a broad linear band with enhanced uptake along the medial tibial periosteum [8]. Recent investigations in athletes with persistent MTSS have demonstrated that it is primarily a bone overload injury. These investigations demonstrated a reduction in bone mineral density of the tibia at the site of MTSS, which subsequently returned to baseline levels upon resolution of MTSS symptoms [9]. Another investigation revealed microcracks without evidence of a repair response in biopsy specimens obtained from symptomatic regions of athletes diagnosed with MTSS, potentially indicating compromised bone repair capacity [10]. Owing to the paucity of substantial evidence supporting theories regarding its pathology, MTSS is predominantly considered a clinical pain syndrome [9].

Clinical presentation

Patients with MTSS frequently present with bilateral tibial aches on the medial side of the middle tibia [11]. However, MTSS may affect the anterior, posterior, or lateral sides of the leg [12]. Pain is induced by physical exertion and alleviated by rest. It is commonly experienced at the onset of an activity and diminishes in magnitude as the exercise progresses. Pain intensity frequently increases the following morning, although it may diminish over time. Resting pain is experienced in patients with extreme and persistent MTSS. Patients have reported pain radiating to the foot and dysesthesia [11]. It is widely accepted that tenderness along the posteromedial tibial border spanning ≥5 cm is indicative of a diagnosis of MTSS, whereas tenderness spanning <5 cm is indicative of SF [13]. Clinically, it is necessary to exclude other pathologies, including tendonitis, chronic exertional compartment syndrome (CECS), vasculitis, nerve impingement, and popliteal artery entrapment, which warrant consideration when evaluating patients with MTSS [14-16].

Yates and White proposed the criteria for the diagnosis of MTSS. Patient symptoms are defined as "pain along the posteromedial border of the tibia that occurs due to EILP from ischemic origin or signs of SF." The diagnosis of MTSS was based on the following criteria: "pain history" as it is induced by exercise and lasts for a few hours or days after exercise; it is not associated with a history of paresthesia or other symptoms indicative of EILP; "pain location" as observed along the posteromedial border of the tibia and the site has to be spread over a minimum distance of 5 cm, whereas focal areas of 2-3 cm are typical of SF; and "palpation" as palpation of the posteromedial border of the tibia produces discomfort that is diffuse and confined to the posteromedial border of the tibia. The bone surface may be uneven in areas of discomfort [13].

Diagnostic studies

Diagnostic imaging techniques can be utilized to evaluate MTSS. X-rays can be used to exclude SF. In addition, a triple-phase bone scan can elucidate the differences between SF and MTSS [7]. In recent years, imaging modalities have transitioned from triple-phase bone scans to MRI because of the superior resolution provided by the MRI technology [17].

Treatment

Various therapeutic interventions have been proposed for patients with MTSS, including rest, cryotherapy, stretching and strengthening exercises, extracorporeal shockwave therapy, stretching and strengthening exercises, graded running programs, gait retraining, lower-leg braces, and injection therapies [9].

Despite the increasing incidence of MTSS, research on its underlying mechanisms remains inadequate. Consequently, it is imperative to elucidate the mechanisms underlying MTSS to develop appropriate prevention strategies based on risk factors and biomechanical disorders. This scoping review aimed to synthesize the characteristics and findings of studies on MTSS, with a particular emphasis on epidemiology, biomechanics, and risk factors. This review elucidates the knowledge gaps pertaining to MTSS and proposes future research directions to enhance our understanding of the biomechanics and risk factors associated with this condition.

## Review

Materials and methods

Study Design

Given the limited and varied literature on MTSS, a scoping review was chosen to comprehensively cover the breadth of existing knowledge on its epidemiology, biomechanics, and risk factors. The scoping review framework created by Arksey and O’Malley was adopted in this review, involving the following phases: formulation of the research question, identification of potentially relevant studies, selection of eligible studies, extraction and organization of data, and summary and reporting of results [18]. Furthermore, we followed the Preferred Reporting Items for Systematic Reviews and Meta-Analyses Extension for Scoping Reviews (PRISMA-ScR) [19].

Objectives

This review aimed to identify the epidemiological profile, biomechanics, and risk factors related to MTSS based on the available evidence.

Literature Search

The search strategy included the following databases: PubMed, Scopus, Cochrane, and Web of Science, from inception to December 31, 2023. The search terms used were "medial tibial stress syndrome" (MeSH) OR "medial tibial periostalgia" OR "shin splints" OR "shin splint" OR "medial tibial stress syndromes" OR "tibial pain" OR "medial tibial periostitis" OR "tibial periostitis". Detailed search strings and filters applied to each database are provided in Appendix A.

Eligibility Criteria

Studies were included if they investigated the epidemiology, biomechanics, and risk factors of adult MTSS patients, were published in English, and focused specifically on MTSS. Exclusion criteria included studies on other causes of tibial pain (e.g., SF, CECS, nerve entrapment, vascular etiologies), patients younger than 18 years, and study types such as case reports, abstracts, protocols, reviews, conference proceedings, opinions/editorials, and consensus statements. Detailed inclusion and exclusion criteria are provided in Appendix B.

Study Selection

The initial search methodology was designed to maximize comprehensiveness and encompass the maximum number of relevant studies. Predetermined inclusion and exclusion criteria were used to refine relevant studies. The study selection process comprised two distinct phases. The initial screening of titles, abstracts, and keywords was conducted in accordance with established inclusion and exclusion criteria. Two reviewers evaluated the papers independently. Studies that did not meet more than one of the inclusion criteria were excluded. Duplicate entries have been eliminated. Full texts deemed eligible for inclusion were obtained, thoroughly examined, and ultimately incorporated if they satisfied all inclusion criteria.

Data Extraction and Synthesis

Data extraction was conducted using a standardized form developed based on the Joanna Briggs Institute (JBI) scoping review methodology [20]. Extracted data included study methodology, objectives, context (setting), sample description, sample size, tools for measuring results, results, conclusions, comments, and issues raised. Two researchers independently extracted data, and discrepancies were resolved through discussion. A narrative synthesis was performed to summarize and elucidate the findings, with key themes and patterns identified and described. Detailed data extraction forms and synthesis methods are provided in Appendix C. A comparison of the characteristics of the studies and the extracted data was performed using tables.

Results

Search Results

**Figure 1:**
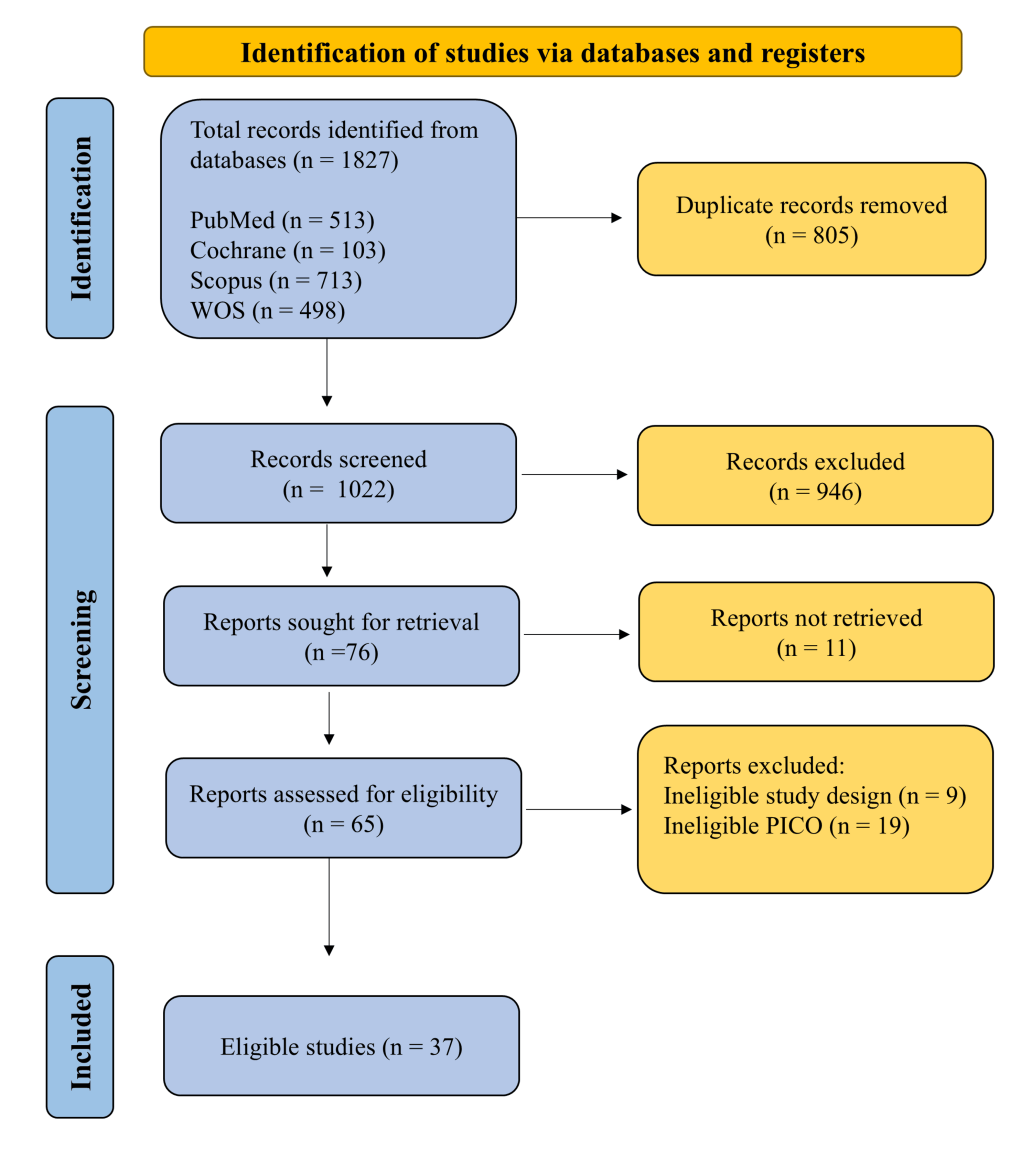
Preferred Reporting Items for Systematic Reviews and Meta-Analyses (PRISMA) flow chart of the search process PICO: Population, Intervention, Comparison, Outcome; WOS: Web of Science

The literature search yielded 1827 records. After removing 805 duplicates, 1022 records were screened by title and abstract, resulting in the exclusion of 946 reports, while 76 reports were sought for retrieval. Only 11 reports were not retrieved. Of the 65 reports assessed for eligibility, nine reports were of an ineligible study design, while 19 had ineligible PICO (Population, Intervention, Comparison, Outcome) elements. Finally, 37 studies were included in this review after fulfilling the inclusion criteria (Figure 1).

Characteristics of Included Studies

**Figure 2:**
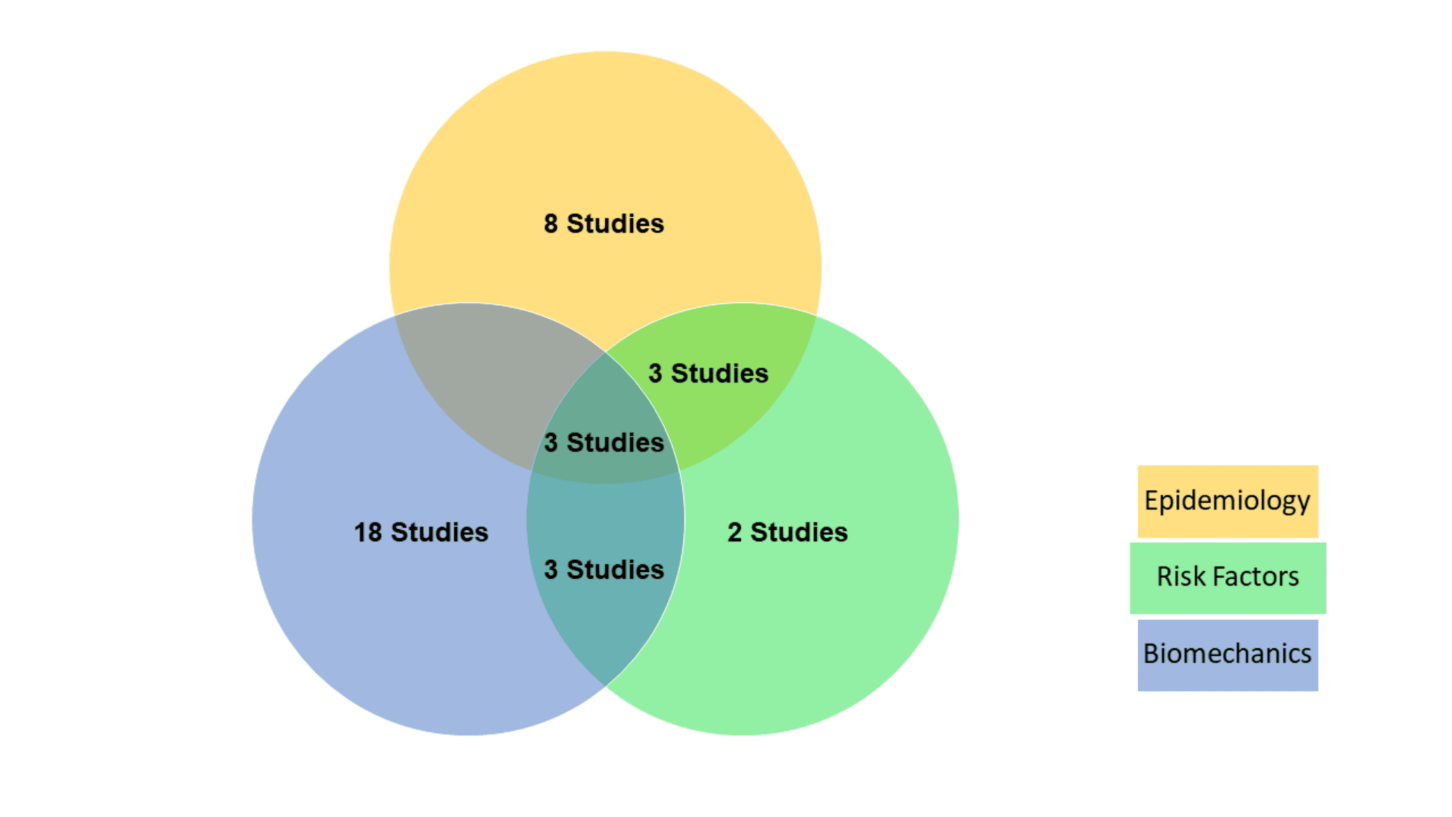
Distribution of studies according to objectives

Of the 37 included articles, eight studies were on epidemiology, two were on MTSS risk factors, and 18 were on biomechanics. Three studies examined both the epidemiology and risk factors of MTTS, three investigated both biomechanics and risk factors, and three investigated epidemiology, risk factors, and biomechanics (Figure 2). The studies encompassed 20 cross-sectional studies, seven cohort studies, five case-control studies, one combined cross-sectional and case-control study, one single-blinded controlled and cross-sectional study, one randomized trial, one controlled laboratory study, and one descriptive laboratory pilot study. Ten articles assessed the prevalence rate of MTSS, and four evaluated its incidence. Six articles examined the risk factors for MTSS among athletes, four evaluated navy subjects, and one evaluated healthy individuals. Fifteen studies evaluated the biomechanics of MTSS among athlete subjects, three evaluated navy subjects, and the rest evaluated cadavers and patients.

**Figure 3:**
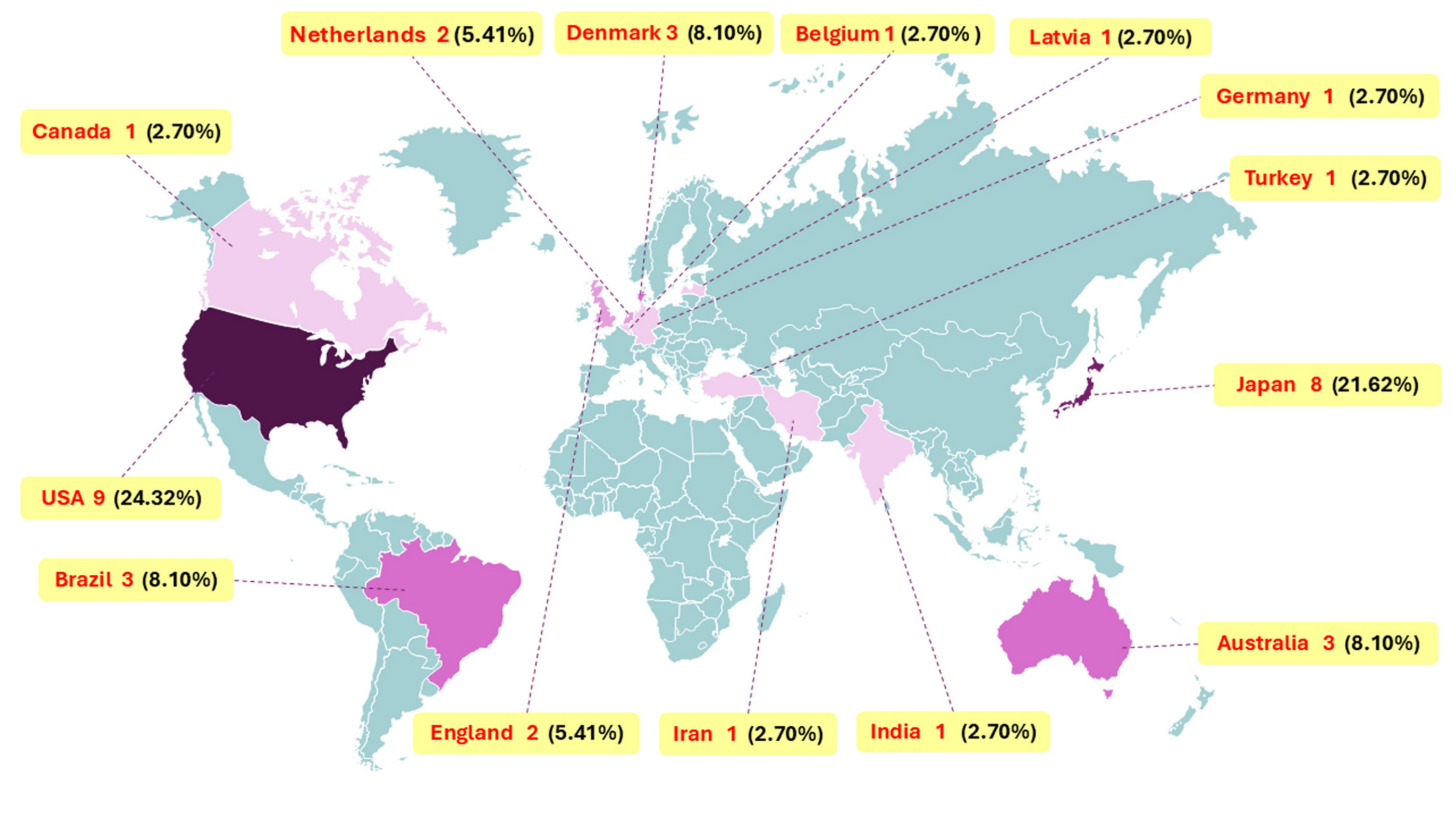
Map visualization showing evidence sources distribution by countries

These studies were conducted in the USA (n = 9) [21-29], Brazil (n = 3) [30-32], Denmark (n = 3) [33-35], England (n = 2) [36,37], the Netherlands (n = 2) [38,39], Japan (n = 8) [40-47], Australia (n = 3) [4,13,48], with one study each from Canada [49], Belgium [50], Latvia [51], Germany [52], Iran [53], Turkey [54], and India [55] (Figure 3). However, no such study has yet been conducted in Africa. Notably, all the published studies included in this scoping review were conducted between 1985 and 2023.

Study Participants

Most of the studies were of mixed gender (62.1%), 21.6% were male, 8.1% were female, and 8.1% were not specified. The vast majority of articles recruited athletes as study participants (67.6%). This was followed by articles about military members (16.2%). Two studies (5.4%) examined cadavers, and two (5.4%) did not specify their subjects. Only one article (2.7%) included a mixed group (cadavers and patients), and another article (2.7%) included a healthy population. The largest sample size was 1368 participants in an American cross-sectional study by Chandran et al. [22]. A descriptive laboratory pilot study in the USA by Bouché and Johnson had the smallest number of subjects, with only three cadavers [29].

Epidemiology of MTSS

The highest prevalence of MTSS has been reported among recreational marathon runners in India (69.5%). The majority of participants were within the age range of 23-25 years (46.3%), with almost equal numbers of male and female participants [55]. The lowest prevalence was reported among American women’s track and field participants (5.4%) [22]. The highest incidence of MTSS was found in a German study that assessed the correlation between acute and overuse injuries and the number of training hours in master-running athletes (35.7%). The majority of the subjects were male (85.8%), belonged to the average age group (42 ± 9 years), and the body mass index (BMI) was male (23.3 ± 2) and female (21.3 ± 2) [52]. The lowest incidence of MTSS (16.07%) was reported in a Danish study, which described the incidence of various running-related injuries among recreational runners and determined their time to recovery (Table 1) [33].

**Table 1:** Studies assessing the epidemiology of MTSS MTSS: medial tibial stress syndrome; NR: not reported

Reference	Design	Country	Participants	Number of participants	Male percentage (%)	Participants' age (years)	BMI (kg/m^2^)	Duration of symptoms	Major findings
Santos et al. (2021) [32]	Descriptive, longitudinal, epidemiological study	Brazil	Athletes	10	100.00	27 ± 5.4	NR	NR	Prevalence of MTSS = 28.6%
Chandran et al. (2021) [21]	Cross-sectional study	USA	Athletes	429	0.00	NR	NR	NR	Prevalence of MTSS = 10.0%
Chandran et al. (2021) [22]	Cross-sectional study	USA	Athletes	1368	0.00	NR	NR	NR	Prevalence of MTSS = 5.4%
De Oliveira et al. (2021) [30]	Observational and cross-sectional study	Brazil	Athletes	195	60.51	37.1 ± 10.7	24.4 ± 2.3	≤ 6 months	MTSS is the primary injury reported in running-related injuries of the lower limbs among recreational street runners. Prevalence of MTSS = 47%.
Fredette et al. (2021) [49]	Cross-sectional study	Canada	Military runners	107	87.85	30.7 ± 8.9	NR	≥ 1 week	Rate of MTSS = 11.2%
Naderi et al. (2020) [39]	A prospective study	Netherlands	Healthy students	123	41.5	MTSS group = 23.1 ± 2.2	MTSS group = 21.5 ± 2.3	Asymptomatic	Incidence of MTSS= 20.5%
Nesterovica et al. (2020) [51]	Cross-sectional study	Latvia	Army	227	93.83	29.6 ± 7.2	NR	NR	Overuse injuries were reported in 43% of cases, and 9.18% of overuse injuries were due to MTSS.
Patel and Patil (2020) [55]	Cross-sectional observational study	India	Athletes	190	50.00	Range: 20 to 30	NR	NR	Prevalence of MTSS in the marathon runners = 69.5%
Wiegand et al. (2019) [25]	Cross-sectional survey	USA	Current and former athletes	312	70.19	38 ± 12	NR	≤ 4 weeks	Prevalence of MTSS = 22%
Garnock et al. (2018) [48]	Prospective cohort study	Australia	Army	123	77.2	NR	NR	≥ 2 weeks	Incidence of MTSS = 24%
Mulvad et al. (2018) [33]	Randomized trial	Denmark	Athletes	839	37.90	39.2 ± 10.0	NR	< 7 days	Incidence proportion of MTSS = 16.07%; time for recovery = 70 days
Sobhani et al. (2015) [53]	Cross-sectional study	Iran	Army	181	100.00	29.52 ± 3.88	19.97 ± 4.83	≥ 2 weeks	Prevalence of MTSS= 16.6%
Knobloch et al. (2008) [52]	Cross-sectional study	Germany	Athletes	291	85.22	42 ± 9	Male: 23.3 ± 2; female: 21.3 ± 2	> 1 day	MTSS rate (number of injuries per 1000 km of running exposure) = (0.01/1000 km); incidence of MTSS = 35.7%
Yates and White (2004) [13]	A prospective study	Australia	Army	124	67.74	MTSS group = 20.95 ± 3.92	MTSS group = 23.95 ± 2.50	≥ 7 days	Incidence of MTTS = 35%

Risk Factors of MTSS

In young-age persons with MTSS, Naderi et al. and Okunuki et al. [39,44] reported that participants with MTSS had greater BMI values than those without MTSS, although a significant correlation between BMI and the time required for complete recovery was established by Moen et al. [38]. A relationship between MTSS and sex has been reported in naval recruits [13,48] and marathon runners [55].

The findings of an American study indicated that those with greater experience in athletic activities are at a lower risk of developing MTSS later in their career [24]. A similar result has been reported in an Indian study [55]. In contrast, a German study found that ultramarathon running increased the incidence of MTSS overuse injuries [52]. Based on the study by Garnock et al. [48], a history of MTSS is the strongest predictor of MTSS development. Naderi et al. indicated that those with a history of MTSS are at a heightened risk of developing MTSS [39]. Additionally, Hubbard et al. found that athletes with a history of MTSS and SF were significantly more prone to experience MTSS [24].

Garnock et al., in their investigation, have discovered that a model that incorporates sex, MTSS history, and hip external rotation demonstrated a high predictive accuracy for identifying individuals at risk of MTSS. The combination of female sex, MTSS history, and hip external rotation yielded a robust and accurate model (82% sensitivity and 84% specificity) for predicting the risk of developing MTSS in navy recruits [48]. 

Franklyn et al. found that individuals with MTSS displayed lesser tibial cross-sectional measurements than their healthy exercising counterparts [4]. A significant difference was observed in the iliospinale height and trochanteric tibial lateral length as anthropometric and anatomical factors between healthy individuals and those diagnosed with MTSS [53]. The iliotibial band (ITB) is an important structure involved in lower-limb movement [56]. Runners who developed MTSS demonstrated a tighter ITB than the uninjured runners (Table 2) [23]. 

**Table 2:** Studies assessing the risk factors for MTSS EMG: electromyography; MTSS: medial tibial stress syndrome; NR: not reported

Reference	Study design	Country	Participants	Number of participants	Male percentage (%)	Participants' age (years)	Duration of symptoms	Assessment tool (diagnostics)	Major findings
Naderi et al. (2020) [39]	A prospective study	Netherlands	Healthy students	123	41.5	MTSS group = 23.1 ± 2.2	Asymptomatic	Dynamic and static foot posture (measuring dynamic arch index (DAI) and foot posture index (FPI)), and soleus and tibialis anterior muscle activity (EMG measurement) during the stance phase of running were measured before a 17-week track and field course.	BMI, MTSS history, and previous vigorous physical activity were predictors of MTSS.
Patel and Patil (2020) [55]	Cross-sectional observational study	India	Athletes	190	26.3	Range: 20 to 30	NR	The runner’s questionnaire	There was a statistically significant association between the MTSS and female sex. In addition, there was a significant association between MTSS and pain throughout running. The occurrence of MTSS was not dependent on the duration of running.
Okunuki et al. (2019) [44]	Cross-sectional study	Japan	Athletes	22	90.9	MTSS group = 20.5 ± 1.5	≥ 2 weeks	Segment angles of the hindfoot and forefoot during walking and running barefoot on a treadmill were recorded using three-dimensional kinematic analysis.	Participants with MTSS exhibited higher BMI values than those without MTSS.
Becker et al. (2018) [23]	A prospective study	USA	Athletes	18	50.0	MTSS group 19 ± 1.07	NR	Clinical examination to assess passive range of motion and muscle strength in the hips and ankles. Plantar pressure analysis was used to quantify mediolateral pressure balance while walking, and 3D motion capture was used to quantify running kinematics.	Runners who developed MTSS demonstrated tighter iliotibial bands.
Garnock et al. (2018) [48]	Prospective cohort study	Australia	Army	123	77.2	NR	≥ 2 weeks	Measures for navicular drop, hip external rotation, and ankle plantarflexion.	A combination of female gender, MTSS history, and hip external rotation provides a strong and accurate predictive model for future risk of developing MTSS in the Navy.
Sobhani et al. (2015) [53]	Cross-sectional study	Iran	Army	181	100.0	29.52 ± 3.88	≥ 2 weeks	Evaluation of leg length discrepancy, the iliospinale height, lateral tibial height, and trochanteric-lateral tibial height were measured based on anthropometric assessments.	Iliospinale height and trochanteric tibial lateral length were significantly different between healthy subjects and patients with MTSS.
Moen et al. (2012) [38]	Case-control study	Netherlands	Army	35	100.00	MTSS group = 19 ± 1.5	≥ 2 weeks	Measurement of hip internal and external ranges of motion, knee flexion and extension, dorsal and plantar ankle flexion, hallux flexion and extension, subtalar eversion and inversion, maximal calf girth, lean calf girth, standing foot angle, and navicular drop test. The range of motion was measured using a goniometer.	BMI was significantly associated with time to full recovery.
Hubbard et al. (2009) [24]	Prospective, multisite, cohort study	USA	Athletes	146	44.5	19.75 ± 1.7	≥ 2 weeks	Physical examination to assess their ankle/foot strength, ankle/foot range of motion, tibial varum, and navicular drop.	A total of 44.6% of the MTSS group members accounted for the following variables: previous history of stress fracture, use of orthotics, running history, and previous history of MTSS. Of these, the most important factors for predicting the development of MTSS were a previous history of MTSS and years of running experience. Collectively, 55.4% of the MTSS group membership remained unexplained.
Franklyn et al. (2008) [4]	Cohort study	Australia	Athletes	88	48.9	MTSS group/males = 22.5 ± 4.2; MTSS group/female = 23.3 ± 3.3 =	NR	Tibial scout radiographs and cross-sectional computed tomography images were obtained at the junction of the mid-third and distal third of the tibia. Tibial dimensions were measured directly from the films, and other parameters were calculated numerically.	MTSS subjects had smaller tibial cross-sectional dimensions than did their uninjured exercising counterparts, suggesting that medial tibial stress syndrome is not just a soft-tissue injury but also a bony injury.
Knobloch et al. (2008) [52]	Cross-sectional study	Germany	Elite running athletes	291	85.2	42 ± 9	> 1 day	Questionnaire	Risk factors: Running more than four times a week or for more than 2600 km exposure had a higher risk for shin splint overuse injuries. Ultra-marathon running increased MTSS overuse injury.
Yates and White (2004) [13]	A prospective study	Australia	Army	124	67.7	MTSS group = 20.95 ± 3.92	≥ 7 days	A questionnaire was used to collect prospective data. Biomechanical assessment, including ankle joint dorsiflexion and foot posture measurements, and FPI parameters, was measured for both feet.	Female sex was a significant risk factor for MTSS.

Biomechanics for MTSS

Tibial fascial tension contributes to MTSS [29]. An earlier study discovered that the soleus muscle and its associated fascia are implicated in tibial stress changes, notably in the pronated heel [28]. A study conducted in 2020 revealed that the MTSS group exhibited a higher peak EMG amplitude in the soleus muscle than did the control group during the absorption and propulsion phases of running. However, no significant difference was detected in the tibialis anterior muscle between the two groups [39].

The deep crural fascia (DCF) attaches to the periosteum at the anterior and medial tibial borders [57]. Stickley et al. assessed the tibial attachments of the DCF, tibialis posterior, soleus, and flexor digitorum longus in relation to the medial malleolus. The investigators of this study discovered that theories concerning traction-induced injuries involving the superficial and deep posterior compartment muscles have not yet been substantiated by anatomical proof. In contrast, the tibial attachments of DCF found in this investigation provide evidence of the involvement of DCF in traction-induced injuries [26].

Noh et al. reported that soccer players with a clinical picture of MTSS had abnormal structural foot deformation during the stance phase of running. They had higher magnitudes of angular change and translational motion of the medial longitudinal arch (MLA) and lateral longitudinal arch (LLA) [45]. Researchers have shown that runners with MTSS experience greater pressure on the medial than on the lateral aspect of their feet during initial contact, foot flat, and heel-off [23]. Moreover, Kinoshita et al. found that the percentage of body weight in the medial metatarsal region is significantly higher in patients with MTSS [46].

The mean navicular drop test (NDT) score was significantly lower in the MTSS group than in the control group in the Sobhani et al. study [53]. According to Moen et al., a positive NDT outcome is significantly correlated with the MTSS [38]. Furthermore, Rathleff et al. observed that individuals diagnosed with MTSS demonstrated greater ND and higher ND velocity while walking on a treadmill [34]. Biomechanical evaluation of the range of motion (ROM) for ankle dorsiflexion [27,36], ankle dorsiflexion excursion [42], and ankle plantar flexion ROM [38] was assessed in four studies. A recent investigation revealed that recreational runners diagnosed with MTSS demonstrated reduced strength in the sagittal plane of the ankle without exhibiting an association with pain intensity or a change in the ratio of plantar flexors to dorsiflexors [31].

Two significant discriminators were identified between the healthy and MTSS groups: maximum pronation velocity and maximum pronation [27]. Tweed et al. also reported a significant relationship between MTSS development and overpronated feet [37]. Researchers have also discovered a significant correlation between the MTSS and talocrural joint equinus, early heel lift, abductory twist, and apropulsive gait while running on a treadmill. In an Australian study, biomechanical evaluation revealed greater foot pronation in the MTSS group [13]. A prospective study showed that dynamic foot pronation is a significant predictor of MTSS occurrence during running. Conversely, Akiyama et al. found no statistically significant differences in the ROM of all ankle joint angles during forward stepping between individuals diagnosed with MTSS and healthy controls [41]. A recent study concluded that while running, the maximum ankle eversion moment during the stance phase was higher in the MTSS group. Even after improvement in MTSS symptoms, the running biomechanics of individuals with a history of MTSS differed from those of the asymptomatic participants [43]. Becker et al. found that athletes who acquired MTSS had peak rearfoot eversion amount and duration during the stance phase [23]. In a study conducted by Okunuki et al., subjects with MTSS demonstrated significantly greater hindfoot eversion and abduction during ambulatory and running activities, forefoot eversion and abduction during ambulation, and forefoot abduction during running compared to those without MTSS [44]. An imbalance in strength between the ankle invertor and evertor muscles in individuals with MTSS may have a role in the etiology of this condition [54]. Akiyama et al. reported a significantly larger range of inversion/eversion and internal/external rotation motion was detected in the talocalcaneal joint of patients with MTSS from heel contact to heel-off phases [41].

A significantly higher percentage of the proximal phase between the rear and midfoot in the sagittal and coronal planes was observed in the group with a history of MTSS [42]. Kinoshita et al., in their investigation, revealed that the leg-heel angle (LHA) during walking was considerably greater in MTSS, whereas the LHA during standing remained unchanged [46]. However, the percentage of the foot width during walking was significantly lower. Patients with MTSS are characterized by an inverse relationship between the complexity of foot kinematics and surface electromyography (SEMG) signals. It has a higher complexity of the SEMG signal of the tibialis anterior and soleus muscles but a lower complexity in midfoot kinematics [35]. No evidence indicated that dynamic loading significantly increased with outdoor running fatigue in runners with a history of MTSS. However, in the MTSS, running fatigue is associated with decreased mediolateral sample entropy [50].

An analysis of injured runners demonstrated greater contralateral pelvic drop [23,36] and forward trunk leaning at midstance in the MTSS group [36]. Hip adduction was greater among the subgroups of runners with MTSS [36], and according to Becker et al. [23], individuals with MTSS displayed a weakness in their hip abductors, as compared to healthy runners. Studies have reported that individuals in the MTSS group exhibit reduced ROM in both internal [38,53] and external [53] hip movements. The shear modulus of the lower leg muscles in patients with MTSS was investigated in two studies (Table 3) [40,47].

**Table 3:** Studies exploring the biomechanics of MTSS MTSS: medial tibial stress syndrome; NR: not reported; SEMG: surface electromyography

Reference	Design	Country	Participants	Number of participants	Male percentage (%)	Participants' age (years)	Duration of symptoms	Assessment tool (diagnostics)	Major findings
Ohmi et al. (2023) [43]	Case-control study	Japan	Athletes	13	100.00	MTSS = 23.600 ± 2.408	≤ 6 months	The participants were assessed using a three-dimensional motion analysis system (eight cameras, two force plates, and 16 infrared reflective markers).	The maximum ankle eversion moment during the stance phase of running was larger in the MTSS group than in the non-MTSS group. Even after the disappearance of MTSS symptoms, the running biomechanics of participants with previous MTSS differed from those of participants without previous MTSS.
Jardim et al. (2022) [31]	Cross-sectional study	Brazil	Athletes	36	44.4	27.6 ± 7.40	≤ 3 months	An isokinetic dynamometer was used to evaluate the peak torque of the ankle dorsiflexors and plantar flexors.	The MTSS group showed lower normalized isokinetic peak torque in the dorsiflexors in the concentric and eccentric contraction, as well as lower plantar flexor and normalized isokinetic peak torque in the concentric and eccentric contraction, compared to the control group. However, there was no difference in the normalized isokinetic peak torque ratio, which was representative of the stance and swing phases.
Akuzawa et al. (2022) [42]	Cross-sectional study	Japan	Athletes	24	0.00	MTSS group = 19.9 ± 1.4	≥ 1 week	Foot kinematics were analyzed using three-dimensional assessments (16 reflective markers, eight infrared cameras, and a force plate), including the angle at the landing and peak angle from landing to leaping and excursion at the rearfoot, midfoot, and forefoot during single leg drop jumps.	The group with a history of MTSS showed a significantly higher percentage of proximal phase between the rear foot and mid-foot in the sagittal and coronal planes. Dorsiflexion excursion was significantly larger in the MTSS history group than in the no-history group.
Naderi et al. (2020) [39]	A prospective study	Netherlands	Healthy students	123	41.5	MTSS group = 23.1 ± 2.2	Asymptomatic	Dynamic and static foot posture (measuring dynamic arch index (DAI) and foot posture index (FPI)), and soleus and tibialis anterior muscle activity (EMG measurement) during the stance phase of running were measured before a 17-week track and field course.	Dynamic foot index and soleus peak EMG amplitude during propulsion were predictors of MTSS.
Kinoshita et al. (2019) [46]	Cross-sectional study	Japan	Athletes	33	60.61	MTSS group = 20.7 ± 1.3	≥ 2 weeks	Foot pressure measurements were performed using a flexible, pressure-sensitive sheet of the foot pressure measurement system (F-Scan II; Nitta Corp., Osaka, Japan), which was used as the insole for the trial shoes (Shin-Nippon Kyoiku shoes). During walking, leg-heel angle (LHA) was continuously recorded using an electronic goniometer.	The LHA while walking was significantly higher in individuals with MTSS, whereas the LHA while standing was not significantly different. The percentage of body weight in the medial metatarsal areas was significantly higher in patients with MTSS, whereas the percentage of foot width on walking was significantly lower.
Okunuki et al. (2019) [44]	Cross-sectional study	Japan	Athletes	22	90.91	MTSS group = 20.5 ± 1.5	≥ 2 weeks	Segment angles of the hindfoot and forefoot during walking and running barefoot on a treadmill were recorded using three-dimensional kinematic analysis.	Subjects with MTSS exhibited significantly greater hindfoot eversion and abduction during walking and running, forefoot eversion and abduction during walking, and forefoot abduction during running than subjects without MTSS.
Becker et al. (2018) [23]	A prospective study	USA	Athletes	18	50.00	MTSS group = 19 ± 1.07	NR	Clinical examination to assess passive range of motion and muscle strength in the hips and ankles. Plantar pressure analysis was used to quantify mediolateral pressure balance while walking, and 3D motion capture was used to quantify running kinematics.	Runners who developed MTSS demonstrated more pressure under the medial aspect of their foot at initial foot contact, foot flat, and heel off, greater peak amounts and durations of rear foot eversion during the stance phase, greater contralateral pelvic drop, and weaker hip abductors.
Bramah et al. (2018) [36]	Controlled laboratory study	England	Athletes	108	39.81	MTSS subgroup = 31.9 ± 9.7	≥ 3 months	Kinematic data were collected using a 12-camera Oqus system. Nine anatomical segments were tracked using markers to track each segment while running on a treadmill.	The subgroup analysis of the injured runners demonstrated greater contralateral pelvic drop and forward trunk lean at midstance, and a more extended knee and dorsiflexed ankle at initial contact were consistent across each of the four injured subgroups, including MTSS. Hip adduction was greater among subgroups of runners with MTSS and patellofemoral pain than in the iliotibial band syndrome subgroup.
Saeki et al. (2018) [47]	Cross-sectional study	Japan	Athletes	24	100.00	20.0 ± 1.7	NR	The shear elastic moduli of the lateral gastrocnemius, medial gastrocnemius, soleus, peroneus longus, peroneus brevis, flexor hallucis longus, flexor digitorum longus, and tibialis posterior were measured using ultrasonic shear wave elastography.	The share elastic moduli of the flexor digitorum longus and tibialis posterior were significantly higher in subjects with a history of MTSS than in those with no history.
Schütte et al. (2018) [50]	Cross-sectional study	Belgium	Athletes	30	60.00	MTSS group = 20.36 ± 0.84	13.9 ± 7.7 months	Accelerometer-based measures included dynamic loading of the trunk and tibia as well as dynamic trunk stability. Regression coefficients from generalized estimating equations were used to evaluate the groups based on fatigue interactions.	No evidence could be found for dynamic loading being higher with outdoor running fatigue in runners with previous MTSS compared with uninjured controls. However, in MTSS only, Running fatigue is associated with a decreased mediolateral sample entropy.
Akiyama et al. (2016) [40]	Cross-sectional study	Japan	Athletes	44	100.00	MTSS group = 21.9 ± 6.4	NR	The shear modulus of the medial head of the gastrocnemius, lateral head of the gastrocnemius, soleus, peroneus longus, and tibialis anterior muscles was measured using shear wave ultrasound elastography.	The shear modulus of the lower leg muscles was significantly greater in patients with MTSS than in healthy patients.
Akiyama et al. (2015) [41]	Cross-sectional study	Japan	Athletes	16	100.00	MTSS group = 21.1 ± 2.1	≥ 2 weeks	Forward-step trials were recorded using cineradiographic images. Geometric bone models of the tibia and talus/calcaneus were created from computed tomography scans of the distal part of one lower limb. Following a combination of approaches, anatomical coordinate systems were embedded in each bone model. The talocrural joint motion and subtalar joint motion were examined.	A significantly larger range of internal/external rotation and inversion/eversion motion was observed in the subtalar joint of patients with MTSS than in healthy controls from heel contact to heel-off. There were no significant differences between MTSS patients and healthy participants in the ranges of all talocrural joint angles during the forward step.
Noh et al. (2015) [45]	Case-control study	Japan	Athletes	10	100.00	MTSS group = 21.4 ± 2.3	≥ 2 weeks	Fluoroscopic imaging was used to investigate bone movement during landing or running.	The magnitude of angular change for the medial longitudinal arch (MLA) and lateral longitudinal arch (LLA) was significantly greater in subjects with MTSS than in the control subjects. The translational motion of the MLA and LLA in the MTSS group was also significantly greater than that in the non-MTSS group. Soccer players with MTSS have an abnormal structural deformation of the foot during the support (or stance) phase of running, with a large decrease in both the MLA and LLA.
Sobhani et al. (2015) [53]	Cross-sectional study	Iran	Army	181	100.00	29.52 ± 3.88	≥ 2 weeks	All angles were measured using a goniometer, and all static measurements of the lower extremities were performed using a tape meter. Evaluation of leg length discrepancy, hip range of motion, intercondylar interval, quadriceps (Q) angle, calf girth, ankle girth, and navicular drop (ND) test assessment. The iliospinale height, lateral tibial height, and trochanteric-lateral tibial height were measured based on anthropometric assessments.	Hip internal and external rotation range of motion, and the score of the ND test, were significantly different between healthy subjects and patients with MTSS.
Rathleff et al. (2012) [34]	Case-control study	Denmark	Athletes	28	71.43	MTSS group = 27.8 ± 8.8	≥ 3 months	ND was evaluated during treadmill walking using a two-dimensional video analysis. Static foot posture, static ND, dynamic ND (dND), and velocity of dND were compared.	Patients with MTSS display a larger ND and a higher ND velocity during treadmill walking.
Moen et al. (2012) [38]	Case-control study	Netherlands	Army	35	100.00	MTSS group = 19 ± 1.5	≥ 2 weeks	Measurement of hip internal and external ranges of motion, knee flexion and extension, dorsal and plantar ankle flexion, hallux flexion and extension, subtalar eversion and inversion, maximal calf girth, lean calf girth, standing foot angle, and ND test. The range of motion was measured using a goniometer.	Increased ankle plantar flexion, decreased internal hip range of motion, and a positive ND test were significantly associated with MTSS.
Yüksel et al. (2011) [54]	Single-blinded, controlled and cross-sectional study	Turkey	Patients diagnosed with MTSS	22	63.64	MTTS group = 23.2 ± 2.9	5.0 ± 2.1 weeks	Detailed Exercise Questionnaire. Isokinetic muscle strength testing was performed at 30°/s and 120°/s to assess the strength of the ankle invertor and evertor muscles. Photographs of the weight-bearing and non-weight-bearing feet were taken to measure MLA deformation and ND.	At 30°/s, the average eversion concentric strength was significantly higher in the MTSS group, and the inversion/eversion strength ratio was significantly higher in the control group. At a velocity of 120°/s, the average concentric eversion strength was significantly higher in the MTSS group.
Rathleff et al. (2011) [35]	Case-control study	Denmark	Patients diagnosed with MTSS	25	0.00	MTTS group = 27.8 ± 8.8	≥ 4 weeks	SEMG from the tibialis anterior and the soleus muscles, as well as mid-foot kinematics, were recorded during 20 consecutive gait cycles.	There is an inverse relationship between the complexity of foot kinematics and the complexity of the SEMG signal in patients suffering from MTSS. Patients with MTSS are characterized by higher complexity of SEMG signal of the tibialis anterior and soleus, but lower complexity in midfoot kinematics.
Stickley et al. (2009) [26]	Cross-sectional study	USA	Cadaver	16	31.3	Mean age at time of death = 82.8 ± 11.1	NR	The cadavers were quantified for the tibial attachments of the deep crural fascia (DCF), soleus, flexor digitorum longus, and tibialis posterior, relative to the medial malleolus.	The tibial attachments of the DCF implicate its involvement in creating MTSS.
Tweed et al. (2008) [37]	Cross-sectional study	England	Athletes	40	57.50	18 to 56	NR	Assessment using the FPI to measure static overpronation. Range of motion assessment at the talocrural joint, as was the range of motion at the first metatarsophalangeal joint, and the angular difference between the neutral and relaxed calcaneal stance positions. Video analysis to identify abnormal or mistimed pronations.	There was a relationship between MTSS and statically overpronated foot, talocrural joint equinus, early heel lift, abductory twist, and apropulsive gait while running on a treadmill. Significant dynamic factors are thought to be compensatory mechanisms for significant static factors. The compensatory dynamic mechanisms (early heel lift and abductory twist) are thought to lead to midtarsal joint pronation and, therefore, apropulsive gait. The apropulsive gait possibly overloads and stresses the medial musculature and fascia of the tibia, accentuating MTSS symptoms.
Bouché and Johnson (2007) [29]	Descriptive laboratory pilot study	USA	Cadaver	3	0.0	Mean age at the time of death = 83.7	NR	The strain was measured in the distal tibial fascia using strain gauges placed in the fascia at the medial tibial crest insertion.	As the tension on the posterior tibial, flexor digitorum longus, and soleus tendons increased, the strain in the tibial fascia increased in a consistent linear manner. Therefore, fascial tension may play a role in MTSS pathophysiology.
Yates and White (2004) [13]	A prospective study	Australia	Army	124	67.7	MTSS group = 20.95 ± 3.92	≥ 7 days	A questionnaire was used to collect prospective data. Biomechanical assessment, including ankle joint dorsiflexion and foot posture measurements, and FPI parameters, was measured for both feet.	Pronated foot type was associated with MTSS.
Messier and Pittala (1988) [27]	Combined cross-sectional and case-control study	USA	Athletes	64	NR	NR	NR	Analysis of biomechanical variables, including time to maximum pronation, total rear foot movement, maximum pronation, and maximum pronation velocity. Anthropometric measurements included footprint test, sit and reach test, plantar and dorsiflexion range of motion, quick tests for hamstring and lower leg flexibility, Q angle measurement, and true and apparent leg lengths. Questionnaires to evaluate training variables.	There were two significant discriminators between the control and MTSS groups: maximum pronation velocity and maximum pronation. The dorsiflexion range of motion was lower in the MTSS group.
Michael and Holder (1985) [28]	Cross-sectional study	USA	Cadavers and patients	25	NR	NR	NR	Anatomical dissection (14 human cadavers), muscle stimulation tests were performed on 10 individuals, including two patients with shin splints, and EMG testing on the medial soleus of five of these individuals, and open biopsy (one patient).	The soleus muscle and its investing fascia are anatomically and biomechanically implicated in the production of these stress changes, particularly when the heel is in the pronated position.

Discussion

This scoping review examined articles on MTSS, analyzing its epidemiology, patient characteristics, and associated lower limb biomechanics and risk factors. It also proposed future research directions.

Epidemiological data are utilized to plan and evaluate strategies for disease prevention and guide the management of patients with existing conditions. Similar to pathology and clinical findings, the epidemiology of a disease is a crucial component of its fundamental characteristics. We noticed a marked difference between the lowest and highest prevalence of MTSS (69.5% and 5.4%, respectively), as recorded in India and the USA [55,22]. This variation shows the impact of different demographics and geographic locations on the development of MTSS. In an American study, injury data were retrieved using the participants' clinical electronic medical record systems [22]. In the Indian investigation, the assessment was conducted based on a runner’s questionnaire filled out by the subjects [55].

Investigating possible factors for MTSS, it is possible that elevated BMI values may increase tibial loading, induce stress during bending and/or bowing [58,59], pronounced cortical bone microdamage [60], and effects on forefoot and hindfoot kinematics [44]. Hence, individuals with an elevated BMI may need to modify their training program to allow for a progressive enhancement in physical activity that enables adaptations in musculoskeletal remodeling, thereby reducing the likelihood of sustaining muscle strain injury from the amplified stress exerted on the kinetic chain [39]. Delayed onset of menarche, menstrual irregularities, reduced bone mineral density, leg length discrepancy, diminished lean mass of the shin, and a low-fat diet were identified as risk factors for tibial injury in females [61]. A substantial number of female participants reported that MTSS occurred when they were required to maintain pace with male recruits while marching or doubling (jogging steps). This may have resulted in excessively strenuous gait alterations characterized by abnormally elongated stride lengths, consequently increasing the risk of developing MTSS [13]. Moreover, the bony cross-sectional area determines its resistance to axial compression and represents the total quantity of bone tissue in the cross-section [62]. The correlation between MTSS and tibial cross-sectional measurements indicates that MTSS is not exclusively a soft tissue condition but also encompasses osseous injury [4].

Running is an affordable and widely practiced form of exercise, particularly among those who do not consider themselves athletes. Notably, running is a fundamental component of many sports and physical activities. According to Hubbard et al., the observation that individuals with greater experience in athletic activities are at a lower risk of developing MTSS later in their careers can be explained by the capacity of the tibia to acclimatize to and accommodate forces resulting from prolonged running [24]. Patients with a history of MTSS experience chronic inflammation of the tibial region as a result of injury, which is exacerbated by repetitive stress during running and hinders healing at the affected site. As evidenced by these data, the findings indicate that athletes who have previously suffered from MTSS or SF are at a higher risk of encountering these issues when they resume their training regimens. Therefore, it is necessary to take a preventative approach by screening athletes’ histories. Furthermore, it is crucial to educate athletes about SF, given its higher prevalence in those with MTSS, and it is essential to inform them about the potential consequences of MTSS not being appropriately treated [24].

Historically, periostitis arising from insertional traction of different muscles and tendons has been the predominant postulated cause of MTSS [29]. Traction force within the connective tissue can be generated by intense muscular activation or force during athletic activities involving running. Traction theory is based on a mechanism whereby specific muscles in the lower leg induce periostitis along their origin or attachment sites to the tibial bone [15]. The augmented strain on the tibial fascia potentially impedes bone remodeling by exerting additional stress on the cortical bone [63].

The NDT is used to evaluate the function of the MLA, which is crucial for examining patients with overuse injuries [64]. This test serves as an indicator of foot pronation [53]. Pronation serves as a vital protective mechanism by enabling the foot to absorb and regulate shock in its lower extremity [65]. Excessive ND may generate traction force during running, which is an intrinsic risk factor for MTSS [38]. Identifying the pronated foot before commencing training may reduce the incidence of MTSS by implementing early intervention strategies to manage excessive pronation [13].

The associated biomechanical disorders of the ankle joint with MTSS substantiate the importance of evaluation and observation of ankle muscle strength in the sagittal plane during rehabilitation management of runners with MTSS, which may be vital for complete recovery from injury and return to sports participation [31].

The authors postulated that amplified dynamic foot pronation might augment tibial strain by increasing the peak soleus muscle activity. This phenomenon could potentially lead to overuse and subsequent development of MTSS if the amplified strain surpasses the structural capacity of the tibial bone. It may be hypothesized that runners may derive benefits from the adjustment of dynamic foot pronation through the utilization of arch-support foot orthoses, potentially mitigating tibial strain [39]. The correlation between MTSS and various aspects of dynamic foot pronation during running, including maximum pronation velocity and maximum pronation, implies that dynamic foot posture is a more reliable indicator of MTSS risk than static foot posture. Significant dynamic factors have been postulated to function as compensatory mechanisms for the significant static factors. Compensatory dynamic mechanisms (early heel lift and abductory twist) are hypothesized to lead to midtarsal joint pronation and result in propulsive gait. A propulsive gait may potentially impose excessive load and stress on the medial tibial musculature and fascia of the tibia, thereby exacerbating MTSS symptoms [37].

Runners with MTSS are also known to exhibit proximal deficits at the hip, as well as distal issues at the foot, as mentioned by Becker et al. [23]. According to Burne et al., a particular mechanism exists whereby individuals with increased hip internal rotation ROM exhibit a distinct pattern of running, which in turn places additional strain on the tibia and could potentially result in MTSS [66]. Notably, the same pathomechanism may be triggered by an increase in external rotation ROM of the hip.

Muscle stiffness can be evaluated using ultrasonic shear-wave elastography. This technique facilitates the quantitative measurement of muscular stiffness in vivo as it calculates the shear elastic modulus from the velocity of the shear wave generated by tissue vibration [67]. In a cadaver study, a significant association was established between the degree of muscle stretching and shear elastic modulus, assessed using shear wave elastography [68]. Moreover, the shear elastic modulus exhibits a strong correlation with the torque experienced during passive joint movement and the magnitude of force production in vivo [69,70]. Consequently, the shear elastic modulus, as determined through shear waves, is considered to be an index of the mechanical properties (i.e., stiffness) of the muscle [67]. Muscle stiffness is commonly mitigated through stretching exercises [71,72], and stretching of the involved muscles may contribute to the prevention of MTSS relapse [47].

Limitations

This comprehensive review aimed to evaluate the current evidence on the epidemiology, risk factors, and biomechanics of MTSS and to propose future research directions to optimize the management of this condition. The literature review was limited by the inclusion of English-language papers. In addition, the included studies exhibited heterogeneity in terms of the types of measured factors and participant characteristics. There was an absence of geographical representation; most studies were conducted in the USA and Europe, with a very low number of studies from Asia (apart from Japan), and no studies were conducted in Africa. Many studies in the current review had small sample sizes.

Future Directions and Recommendations

We propose several recommendations for better understanding the epidemiology, risk factors, and biomedical factors associated with MTSS. More standardized measurement outcomes are needed to provide consistency across different studies and populations and to make it easier to compare results and draw general conclusions. It would be advantageous to conduct studies with larger sample sizes to ensure robustness and generalizability of the results. There is a need for more research from different countries to overcome the lack of diversity in geographic representation, which will improve the generalizability of the findings, as cultural, social, and economic factors can differ significantly across regions. Future reviews that include younger age groups will provide a more comprehensive understanding of the MTSS.

## Conclusions

This review demonstrates that MTSS development is associated with multiple epidemiological, risk, and biomechanical factors. However, owing to the substantial variability among studies, further studies are necessary to validate these findings and to identify at-risk populations. These efforts will facilitate the development of effective management and curative intervention strategies targeting specific populations.

## Appendices

Appendix A

**Table 4:** Scoping review search strategies

Database	Search strategies
PubMed	("medial tibial stress syndrome" (MeSH) OR "medial tibial periostalgia" OR "shin splints" OR "shin splint" OR "medial tibial stress syndromes" OR "tibial pain" OR "medial tibial periostitis" OR "tibial periostitis")
Scopus	TITLE-ABS-KEY("medial tibial stress syndrome") OR TITLE-ABS-KEY("medial tibial periostalgia") OR TITLE-ABS-KEY("shin splints") OR TITLE-ABS-KEY("shin splint") OR TITLE-ABS-KEY("medial tibial stress syndromes") OR TITLE-ABS-KEY("tibial pain") OR TITLE-ABS-KEY("medial tibial periostitis") OR TITLE-ABS-KEY("tibial periostitis")
Cochrane	("medial tibial stress syndrome" OR "medial tibial periostalgia" OR "shin splints" OR "shin splint" OR "medial tibial stress syndromes" OR "tibial pain" OR "medial tibial periostitis" OR "tibial periostitis")
Web of Science	("medial tibial stress syndrome" OR "medial tibial periostalgia" OR "shin splints" OR "shin splint" OR "medial tibial stress syndromes" OR "tibial pain" OR "medial tibial periostitis" OR "tibial periostitis")

Appendix B

**Table 5:** Inclusion and exclusion criteria CECS: chronic exertional compartment syndrome; MTSS: medial tibial stress syndrome; TSF: tibial stress fracture

Inclusion criteria	Exclusion criteria
Human studies (including observational studies)	Patients younger than 18 years
Studies that investigated the epidemiology, biomechanics, and risk factors of adult patients with MTSS	Other causes of tibial pain, including TSF, CECS, and vascular etiologies (e.g., functional popliteal artery entrapment syndrome and peripheral arterial disease)
All ethnic groups	
All geographical areas	Case reports, reviews, conference proceedings, abstracts, opinions/editorials, protocols, and consensus statements
Published in English or translated into English	
Studies: inception through 31 December 2023	Incomplete studies (no results)

Appendix C 

**Table 6:** Detailed data extraction form BMI: body mass index; DAI: dynamic arch index; DCF: deep crucial fascia; EMG: electromyography; FPI: foot posture index; LHA: leg-heel angle; LLA: lateral longitudinal arch; MLA: medial longitudinal arch; MTSS: medial tibial stress syndrome; ND: navicular drop; NR: not reported; Q: quadriceps; SEMG: surface electromyography

	Reference	Variable	Findings
1	Santos et al. (2021) [32]	Design	Descriptive, longitudinal, epidemiological study
		Country	Brazil
		Type of participants	5-a-side soccer athletes
		Participants' age (years)	27 ± 5.4
		BMI (kg/m^2^)	NR
		Height (m)	1.71 ± 0.06
		Duration of symptoms	NR
		Number of participants	10
		Male percentage (%)	100.00%
		Assessment tool (diagnostics)	Sports injuries protocol for Paralympic sports in questionnaire form
		Major findings	Prevalence of MTSS = 28.6%
2	Mulvad et al. (2018 [33]	Design	Randomized trial
		Country	Denmark
		Type of participants	Runners
		Participants' age (years)	39.2 ± 1
		BMI (kg/m^2^)	NR
		Height (m)	NR
		Duration of symptoms	< 7 days
		Number of participants	839
		Male percentage (%)	37.90%
		Assessment tool (diagnostics)	Online questionnaire based on the Oslo Sports Trauma Research Center Questionnaire
		Major findings	Incidence proportion of MTSS = 16.07%. Time for recovery, 70 days
3	Chandran et al. (2021) [21]	Design	Cross-sectional study
		Country	USA
		Type of participants	Women’s cross-country athletes
		Participants' age (years)	NR
		BMI (kg/m^2^)	NR
		Height (m)	NR
		Duration of symptoms	NR
		Number of participants	429
		Male percentage (%)	0.00%
		Assessment tool (diagnostics)	Review of clinical electronic medical record systems
		Major findings	Prevalence of MTSS = 10.0%
4	Chandran et al. (2021) [22]	Design	Cross-sectional study
		Country	USA
		Type of participants	Women’s track and field athletes
		Participants' age (years)	NR
		BMI (kg/m^2^)	NR
		Height (m)	NR
		Duration of symptoms	NR
		Number of participants	1368
		Male percentage (%)	0.00%
		Assessment tool (diagnostics)	Review of clinical electronic medical record systems
		Major findings	Prevalence of MTSS = 5.4%
5	De Oliveira et al. (2021) [30]	Design	Observational and cross-sectional study
		Country	Brazil
		Type of participants	Street runners
		Participants' age (years)	37.1 ± 10.7
		BMI (kg/m^2^)	24.4 ± 2.3 kg/m^2^
		Height (m)	1.70 ± 0.6
		Duration of symptoms	≤ 6 months
		Number of participants	195
		Male percentage (%)	60.51%
		Assessment tool (diagnostics)	Self-reported morbidity survey
		Major findings	MTSS is the primary injury reported in running-related injuries of the lower limbs among recreational street runners. Prevalence of MTSS = 47%.
6	Wiegand et al. (2019) [25]	Design	Cross-sectional survey
		Country	USA
		Type of participants	Current and former runners.
		Participants' age (years)	38 ± 12
		BMI (kg/m^2^)	NR
		Height (m)	NR
		Duration of symptoms	≤ 4 weeks
		Number of participants	312
		Male percentage (%)	70.19%
		Assessment tool (diagnostics)	Online survey
		Major findings	Incidence of MTSS = 22%
7	Nesterovica et al. (2020) [51]	Design	Cross-sectional study
		Country	Latvia
		Type of participants	Infantry soldiers
		Participants' age (years)	29.6 ± 7.2
		BMI (kg/m^2^)	NR
		Height (m)	NR
		Duration of symptoms	NR
		Number of participants	227
		Male percentage (%)	93.83%
		Assessment tool (diagnostics)	Survey
		Major findings	Overuse injuries were reported in 43% of cases, and 9.18% of overuse injuries were due to MTSS.
8	Fredette et al. (2021)[49]	Design	Cross-sectional study
		Country	Canada
		Type of participants	Military runners
		Participants' age (years)	30.7 ± 8.9
		BMI (kg/m^2^)	NR
		Height (m)	175.8 ± 8.4 cm
		Duration of symptoms	≥ 1 week
		Number of participants	107
		Male percentage (%)	87.85%
		Assessment tool (diagnostics)	Questionnaire to assess the demographics, personal characteristics, injury location and diagnosis, pain and function, running parameters, and other training characteristics.
		Major findings	Rate of MTSS = (11.2%)
9	Knobloch et al. (2008) [52]	Design	Cross-sectional study
		Country	Germany
		Type of participants	Elite running athletes
		Participants' age (years)	42 ± 9
		BMI (kg/m^2^)	Male: 23.3 ± 2 kg/m^2^ Female: 21.3 ± 2 kg/m^2^
		Height (m)	178 ± 7 cm
		Duration of symptoms	>1 day
		Number of participants	291
		Male percentage (%)	85.22%
		Assessment tool (diagnostics)	Questionnaire
		Major findings	MTSS rate (number of injuries per 1000 km of running exposure) = (0.01/1000 km). Incidence of MTSS = 35.7%. Running more than four times a week or for more than 2600 km exposure had a higher risk for MTSS overuse injuries. Ultra-marathon running increased MTSS overuse injury.
10	Yates and White (2004) [13]	Design	A prospective study
		Country	Australia
		Type of participants	Army
		Participants' age (years)	MTSS group = 20.95 ± 3.92
		BMI (kg/m^2^)	MTSS group = 23.95 ± 2.50 kg/m^2^
		Height (m)	-
		Duration of symptoms	≥ 7 days
		Number of participants	124
		Male percentage (%)	67.74%
		Assessment tool (diagnostics)	A questionnaire was used to collect prospective data. Biomechanical assessment, including ankle joint dorsiflexion and foot posture measurements, and foot posture index parameters were measured for both feet.
		Major findings	Incidence of MTTS = 35%. Female sex was a significant risk factors for MTSS. Pronated foot type was associated with MTSS.
11	Sobhani et al. (2015) [53]	Design	Cross-sectional study
		Country	Iran
		Type of participants	Army
		Participants' age (years)	29.52 ± 3.88
		BMI (kg/m^2^)	19.97 ± 4.83 kg/m^2^
		Height (m)	173.76 ± 6.58 cm
		Duration of symptoms	≥ 2 weeks
		Number of participants	181
		Male percentage (%)	100.00%
		Assessment tool (diagnostics)	History taking and physical examination. All angles were measured using a goniometer, and all static measurements of the lower extremities were performed using a tape meter. Evaluation of leg length discrepancy, hip range of motion, intercondylar interval, quadriceps (Q) angle, calf girth, ankle girth, and navicular drop test assessment. The iliospinale height, lateral tibial height, and trochanteric-lateral tibial height were measured based on anthropometric assessments.
		Major findings	Prevalence of MTSS = 16.6%. Hip internal and external rotation range of motion, iliospinale height, the score of the navicular drop test, and the trochanteric tibial lateral length were significantly different between healthy subjects and patients with MTSS.
12	Patel and Patil (2020)[55]	Design	Cross-sectional observational study
		Country	India
		Type of participants	Recreational marathon runners
		Participants' age (years)	Range 20 to 30
		BMI (kg/m^2^)	NR
		Height (m)	NR
		Duration of symptoms	NR
		Number of participants	190
		Male percentage (%)	50.00%
		Assessment tool (diagnostics)	The runner’s questionnaire
		Major findings	The prevalence of MTSS in the marathon runners = 69.5%. There was a statistically significant association between the MTSS and female sex. In addition, there was a significant association between MTSS and pain during running, and a significant association between MTSS and pain throughout running. The occurrence of MTSS was not dependent on the duration of running.
13	Naderi et al. (2020) [39]	Design	A prospective study
		Country	Netherlands
		Type of participants	Healthy students
		Participants' age (years)	MTSS group = 23.1 ± 2.2
		BMI (kg/m^2^)	MTSS group = 21.5 ± 2.3 kg/m^2^
		Height (m)	MTSS group = 172 ± 4 cm
		Duration of symptoms	Asymptomatic
		Number of participants	123
		Male percentage (%)	41.50%
		Assessment tool (diagnostics)	Dynamic and static foot posture (measuring dynamic arch index (DAI) and foot posture index (FPI)), and soleus and tibialis anterior muscle activity (EMG measurement) during the stance phase of running were measured before a 17-week track- and field-course.
		Major findings	Incidence of MTSS = 20.5%. BMI, dynamic foot index, soleus peak EMG amplitude during propulsion, MTSS history, and previous vigorous physical activity were predictors of MTSS.
14	Akiyama et al. (2016) [40]	Design	Cross-sectional study
		Country	Japan
		Type of participants	Athletes
		Participants' age (years)	MTSS group = 21.9 ± 6.4
		BMI (kg/m^2^)	NR
		Height (m)	MTSS group = 172.1 ± 4.7 cm
		Duration of symptoms	NR
		Number of participants	44
		Male percentage (%)	100.00%
		Assessment tool (diagnostics)	The shear modulus of the medial head of the gastrocnemius, lateral head of the gastrocnemius, soleus, peroneus longus, and tibialis anterior muscles were measured using shear wave ultrasound elastography.
		Major findings	The shear modulus of the lower leg muscles was significantly greater in patients with MTSS than in healthy patients.
15	Saeki et al. (2018) [47]	Design	Cross-sectional study
		Country	Japan
		Type of participants	Collegiate runners
		Participants' age (years)	20.0 ± 1.7
		BMI (kg/m^2^)	NR
		Height (m)	172.7 ± 4.8 cm
		Duration of symptoms	NR
		Number of participants	24
		Male percentage (%)	100.00%
		Assessment tool (diagnostics)	The shear elastic moduli of the lateral gastrocnemius, medial gastrocnemius, soleus, peroneus longus, peroneus brevis, flexor hallucis longus, flexor digitorum longus, and tibialis posterior were measured using ultrasonic shear wave elastography.
		Major findings	The share elastic moduli of the flexor digitorum longus and tibialis posterior were significantly higher in subjects with a history of MTSS than in those with no history.
16	Michael and Holder (1985) [28]	Design	Cross-sectional study
		Country	USA
		Type of participants	Cadavers and patients
		Participants' age (years)	NR
		BMI (kg/m^2^)	NR
		Height (m)	NR
		Duration of symptoms	NR
		Number of participants	25
		Male percentage (%)	NR
		Assessment tool (diagnostics)	Anatomical dissection (14 human cadavers), muscle stimulation tests were performed on 10 individuals, including two patients with shin splints, and EMG testing on the medial soleus of five of these individuals, and open biopsy (one patient).
		Major findings	Histologically, the increased metabolic activity manifested on the radionuclide scan is due to a periostitis with new bone formation. The soleus muscle and its investing fascia are anatomically and biomechanically implicated in the production of these stress changes, particularly when the heel is in the pronated position.
17	Hubbard et al. (2009) [24]	Design	Prospective, multisite, cohort study
		Country	USA
		Type of participants	Healthy collegiate athletes
		Participants' age (years)	19.75 ± 1.7
		BMI (kg/m^2^)	NR
		Height (m)	172.52 ± 10.1 cm
		Duration of symptoms	≥ 2 weeks
		Number of participants	146
		Male percentage (%)	44.50%
		Assessment tool (diagnostics)	Physical examination to assess their ankle/foot strength, ankle/foot range of motion, tibial varum, and navicular drop.
		Major findings	A total of 44.6% of the MTSS group members accounted for the following variables: previous history of stress fracture, use of orthotics, running history, and previous history of MTSS. Of these, the most important factors for predicting the development of MTSS were a previous history of MTSS and years of running experience. Collectively, 55.4% of the MTSS group membership remained unexplained.
18	Stickley et al. (2009)[26]	Design	Cross-sectional study
		Country	USA
		Type of participants	Cadavers
		Participants' age (years)	Mean age at time of death = 82.8 ± 11.1
		BMI (kg/m^2^)	NR
		Height (m)	NR
		Duration of symptoms	NR
		Number of participants	16
		Male percentage (%)	31.30%
		Assessment tool (diagnostics)	The cadavers were quantified for the tibial attachments of the DCF, soleus, flexor digitorum longus, and tibialis posterior, relative to the medial malleolus.
		Major findings	The tibial attachments of the DCF implicate its involvement in creating MTSS.
19	Bouché and Johnson (2007) [29]	Design	Descriptive laboratory pilot study
		Country	USA
		Type of participants	Cadavers
		Participants' age (years)	Mean age at time of death = 83.7
		BMI (kg/m^2^)	NR
		Height (m)	NR
		Duration of symptoms	NR
		Number of participants	3
		Male percentage (%)	0.0%
		Assessment tool (diagnostics)	The strain was measured in the distal tibial fascia using strain gauges placed in the fascia at the medial tibial crest insertion.
		Major findings	As the tension on the posterior tibial, flexor digitorum longus, and soleus tendons increased, the strain in the tibial fascia increased in a consistent linear manner. Therefore, fascial tension may play a role in MTSS pathophysiology.
20	Garnock et al. (2018) [48]	Design	Prospective cohort study
		Country	Australia
		Type of participants	Navy recruits.
		Participants' age (years)	MTSS group = 19 ± 1.5
		BMI (kg/m^2^)	NR
		Height (m)	NR
		Duration of symptoms	≥ 2 weeks
		Number of participants	123
		Male percentage (%)	77.20%
		Assessment tool (diagnostics)	Measures for navicular drop, hip external rotation, and ankle plantarflexion.
		Major findings	Incidence of MTSS = 24%. A combination of female gender, MTSS history, and hip external rotation provides a strong and accurate predictive model for future risk of developing MTSS in Navy recruits.
21	Moen et al. (2012) [38]	Design	Case-control study
		Country	Netherlands
		Type of participants	Army
		Participants' age (years)	MTSS group = 19 ± 1.5
		BMI (kg/m^2^)	MTSS group = 23.8 ± 2.0 kg/m^2^
		Height (m)	MTSS group = 183 ± 6.5 cm
		Duration of symptoms	≥ 2 weeks
		Number of participants	35
		Male percentage (%)	100.00%
		Assessment tool (diagnostics)	Measurement of hip internal and external ranges of motion, knee flexion and extension, dorsal and plantar ankle flexion, hallux flexion and extension, subtalar eversion and inversion, maximal calf girth, lean calf girth, standing foot angle, and navicular drop test. Range of motion was measured using a goniometer.
		Major findings	Increased ankle plantar flexion, decreased internal hip range of motion, and a positive navicular drop test were significantly associated with MTSS. BMI was significantly associated with time to full recovery.
22	Jardim et al. (2022) [31]	Design	Cross-sectional study
		Country	Brazil
		Type of participants	Recreational runners
		Participants' age (years)	27.6 ± 7.40
		BMI (kg/m^2^)	MTSS group = 24.1 ± 2.74 kg/m^2^
		Height (m)	MTSS group = 1.67 ± 0.09 cm
		Duration of symptoms	≤ 3 months
		Number of participants	36
		Male percentage (%)	44.40%
		Assessment tool (diagnostics)	An isokinetic dynamometer was used to evaluate the peak torque of the ankle dorsiflexors and plantar flexors.
		Major findings	The MTSS group showed lower normalized isokinetic peak torque in the dorsiflexors in the concentric and eccentric contraction, as well as lower plantar flexor and normalized isokinetic peak torque in the concentric and eccentric contraction, compared to the control group. However, there was no difference in the normalized isokinetic peak torque ratio, representative of the stance and swing phases.
23	Franklyn et al. (2008) [4]	Design	Cohort study
		Country	Australia
		Type of participants	Athletes
		Participants' age (years)	MTSS group/ males = 22.5 ± 4.2 MTSS group/females = 23.3 ± 3.3
		BMI (kg/m^2^)	MTSS group/ males = 24.0 ± 2.5 kg/m^2^ MTSS group/females = 23.0 ± 2.4 kg/m^2^
		Height (m)	MTSS group/ males = 176.8 ± 6.5 cm MTSS group/females = 163.1 ± 4.1 cm
		Duration of symptoms	NR
		Number of participants	88
		Male percentage (%)	48.90%
		Assessment tool (diagnostics)	Tibial scout radiographs and cross-sectional computed tomography images were obtained at the junction of the mid-third and distal third of the tibia. Tibial dimensions were measured directly from the films, and other parameters were calculated numerically.
		Major findings	MTSS subjects had smaller tibial cross-sectional dimensions than did their uninjured exercising counterparts, suggesting that medial tibial stress syndrome is not just a soft-tissue injury but also a bony injury.
24	Messier and Pittala (1988) [27]	Design	Combined cross-sectional and case-control study
		Country	USA
		Type of participants	Competitive and recreational runners
		Participants' age (years)	NR
		BMI (kg/m^2^)	NR
		Height (m)	NR
		Duration of symptoms	NR
		Number of participants	64
		Male percentage (%)	NR
		Assessment tool (diagnostics)	Analysis of biomechanical variables, including time to maximum pronation, total rear foot movement, maximum pronation, and maximum pronation velocity. Anthropometric measurements included footprint test, sit and reach test, plantar and dorsiflexion range of motion, quick tests for hamstring and lower leg flexibility, Q angle measurement, and true and apparent leg lengths. Questionnaires to evaluate training variables.
		Major findings	There were two significant discriminators between the control and MTSS groups: maximum pronation velocity and maximum pronation. The dorsiflexion range of motion was lower in the MTSS group.
25	Bramah et al. (2018) [36]	Design	Controlled laboratory study
		Country	England
		Type of participants	Runners
		Participants' age (years)	MTSS subgroup = 31.9 ± 9.7
		BMI (kg/m^2^)	MTSS group = 22.2 ± 2.3 kg/m^2^
		Height (m)	MTSS group = 167.3 ± 8.1 cm
		Duration of symptoms	≥ 3 months
		Number of participants	Total = 108 MTSS group = 18
		Male percentage (%)	39.81%
		Assessment tool (diagnostics)	Kinematic data were collected using a 12-camera Oqus system. Nine anatomical segments were tracked using markers to track each segment while running on a treadmill.
		Major findings	The subgroup analysis of the injured runners demonstrated greater contralateral pelvic drop and forward trunk lean at midstance, and a more extended knee and dorsiflexed ankle at initial contact were consistent across each of the four injured subgroups, including MTSS. Hip adduction was greater among subgroups of runners with MTSS and patellofemoral pain than in the iliotibial band syndrome subgroup.
26	Yüksel et al. (2011) [54]	Design	Single-blinded, controlled, and cross-sectional study
		Country	Turkey
		Type of participants	Patients diagnosed with MTSS
		Participants' age (years)	MTTS group = 23.2 ± 2.9
		BMI (kg/m^2^)	MTTS group = 22.0 ± 3.1 kg/m^2^
		Height (m)	MTTS group = 1.73 ± 0.087 cm
		Duration of symptoms	5.0 ± 2.1 weeks
		Number of participants	22
		Male percentage (%)	63.64
		Assessment tool (diagnostics)	Detailed Exercise Questionnaire. Isokinetic muscle strength testing was performed at 30°/s and 120°/s to assess the strength of the ankle invertor and evertor muscles. Photographs of the weight-bearing and non-weight-bearing feet were taken to measure medial longitudinal arch deformation and navicular drop.
		Major findings	At 30°/s, the average eversion concentric strength was significantly higher in the MTSS group, and the inversion/eversion strength ratio was significantly higher in the control group. At a velocity of 120°/s, the average concentric eversion strength was significantly higher in the MTSS group.
27	Rathleff et al. (2011) [35]	Design	Case-control study
		Country	Denmark
		Type of participants	Patients diagnosed with MTSS
		Participants' age (years)	MTTS group = 27.8 ± 8.8
		BMI (kg/m^2^)	MTTS group = 25.8 ± 5.1 kg/m^2^
		Height (m)	MTTS group = 172.7 ± 10.4 cm
		Duration of symptoms	≥4 weeks
		Number of participants	25
		Male percentage (%)	0.00%
		Assessment tool (diagnostics)	SEMG from the tibialis anterior and the soleus muscles, as well as mid-foot kinematics, were recorded during 20 consecutive gait cycles.
		Major findings	There is an inverse relationship between the complexity of foot kinematics and the complexity of the SEMG signal in patients suffering from MTSS. Patients with MTSS are characterized by higher complexity of SEMG signal of the tibialis anterior and soleus, but lower complexity in midfoot kinematics.
28	Schütte et al. (2018) [50]	Design	Cross-sectional study
		Country	Belgium
		Type of participants	Runners
		Participants' age (years)	MTSS group = 20.36 ± 0.84
		BMI (kg/m^2^)	MTSS group = 21.72 ± 1.22 kg/m^2^
		Height (m)	MTSS group = 176.93 ± 10.03 cm
		Duration of symptoms	13.9 ± 7.7 months
		Number of participants	30
		Male percentage (%)	60.00%
		Assessment tool (diagnostics)	Accelerometer-based measures included dynamic loading of the trunk and tibia as well as dynamic trunk stability. Regression coefficients from generalized estimating equations were used to evaluate the groups based on fatigue interactions.
		Major findings	No evidence could be found for dynamic loading being higher with outdoor running fatigue in runners with previous MTSS compared with uninjured controls. However, in MTSS only, Running fatigue is associated with a decreased mediolateral sample entropy.
29	Rathleff et al. (2012) [34]	Design	Cross-sectional study
		Country	Denmark
		Type of participants	Athletes
		Participants' age (years)	MTTS group = 27.8 ± 8.8
		BMI (kg/m^2^)	MTTS group = 25.8 ± 5.1 kg/m^2^
		Height (m)	MTTS group = 172.7 ± 10.4 cm
		Duration of symptoms	≥ 3 months
		Number of participants	28
		Male percentage (%)	71.43
		Assessment tool (diagnostics)	Navicular drop (ND) was evaluated during treadmill walking by a two-dimensional video analysis. Static foot posture, static ND, dynamic ND (dND), and velocity of dND were compared.
		Major findings	Patients with MTSS display a larger ND and a higher ND velocity during treadmill walking.
30	Akuzawa et al. (2022) [42]	Design	Cross-sectional study
		Country	Japan
		Type of participants	Lacrosse players
		Participants' age (years)	MTSS group = 19.9 ± 1.4
		BMI (kg/m^2^)	MTSS group = 19.9 ± 1.4 kg/m^2^
		Height (m)	MTSS group = 161.6 ± 4.2 cm
		Duration of symptoms	≥ 1 week
		Number of participants	24
		Male percentage (%)	0.00%
		Assessment tool (diagnostics)	Foot kinematics were analyzed using three-dimensional assessments (Sixteen reflective markers, eight infrared cameras, and a force plate), including the angle at the landing and peak angle from landing to leaping and excursion at the rearfoot, midfoot, and forefoot during single-leg drop jumps.
		Major findings	The group with a history of MTSS showed a significantly higher percentage of proximal phase between the rear foot and mid-foot in the sagittal and coronal planes. Dorsiflexion excursion was significantly larger in the MTSS history group than in the no-history group.
31	Ohmi et al. (2023) [43]	Design	Case-control study
		Country	Japan
		Type of participants	Long-distance runners
		Participants' age (years)	MTSS = 23.600 ± 2.408
		BMI (kg/m^2^)	MTSS group = 18.874 ± 0.827 kg/m^2^
		Height (m)	MTSS group = 171.7 ± 5.805 cm
		Duration of symptoms	≤ 6 months
		Number of participants	13
		Male percentage (%)	100.00%
		Assessment tool (diagnostics)	The participants were assessed using a three-dimensional motion analysis system (eight cameras, two force plates, and sixteen infrared reflective markers).
		Major findings	The maximum ankle eversion moment during the stance phase of running was larger in the MTSS group than in the non-MTSS group. Even after the disappearance of MTSS symptoms, the running biomechanics of participants with previous MTSS differed from those of participants without previous MTSS.
32	Akiyama et al. (2016) [40]	Design	Cross-sectional study
		Country	Japan
		Type of participants	Soccer players
		Participants' age (years)	MTSS group = 21.1 ± 2.1
		BMI (kg/m^2^)	NR
		Height (m)	MTSS group = 174.6 ± 6.7 cm
		Duration of symptoms	≥ 2 weeks
		Number of participants	16
		Male percentage (%)	100.00%
		Assessment tool (diagnostics)	Forward-step trials were recorded using cineradiographic images. Geometric bone models of the tibia and talus/calcaneus were created from computed tomography scans of the distal part of one lower limb. Following a combination of approaches, anatomical coordinate systems were embedded in each bone model. The talocrural joint motion and subtalar joint motion were examined.
		Major findings	A significantly larger range of internal/external rotation and inversion/eversion motion was observed in the subtalar joint of patients with MTSS than in healthy controls from heel contact to heel-off. There were no significant differences between MTSS patients and healthy participants in the ranges of all talocrural joint angles during the forward step.
33	Kinoshita et al. (2019) [46]	Design	Cross-sectional study
		Country	Japan
		Type of participants	Athletes
		Participants' age (years)	MTSS group = 20.7 ± 1.3
		BMI (kg/m^2^)	MTSS group = 20.8 ± 1.6 kg/m^2^
		Height (m)	MTSS group = 169 ± 5.8 cm
		Duration of symptoms	≥ 2 weeks
		Number of participants	33
		Male percentage (%)	60.61%
		Assessment tool (diagnostics)	Foot pressure measurements were performed using a flexible, pressure-sensitive sheet of the foot pressure measurement system (F-Scan II; Nitta Corp., Osaka, Japan), which was used as the insole for the trial shoes (Shin-Nipponkyoiku shoes). During walking, leg-heel angle (LHA) was continuously recorded using an electronic goniometer.
		Major findings	The LHA while walking was significantly higher in individuals with MTSS, whereas the LHA while standing was not significantly different. The percentage of body weight in the medial metatarsal areas was significantly higher in patients with MTSS, whereas the percentage of foot width on walking was significantly lower.
34	Tweed et al. (2008) [37]	Design	Cross-sectional study
		Country	England
		Type of participants	Runners
		Participants' age (years)	18 to 56
		BMI (kg/m^2^)	NR
		Height (m)	NR
		Duration of symptoms	NR
		Number of participants	40
		Male percentage (%)	57.50%
		Assessment tool (diagnostics)	Assessment using the FPI to measure static overpronation. Range of motion assessment at the talocrural joint, as was the range of motion at the first metatarsophalangeal joint, and the angular difference between the neutral and relaxed calcaneal stance positions. Video analysis to identify abnormal or mistimed pronations.
		Major findings	There was a relationship between MTSS and statically overpronated foot, talocrural joint equinus, early heel lift, abductory twist, and apropulsive gait while running on a treadmill. Significant dynamic factors are thought to be compensatory mechanisms for significant static factors. The compensatory dynamic mechanisms (early heel lift and abductory twist) are thought to lead to midtarsal joint pronation and, therefore, apropulsive gait. The apropulsive gait possibly overloads and stresses the medial musculature and fascia of the tibia, accentuating MTSS symptoms.
35	Becker et al. (2018) [23]	Design	Prospective Study
		Country	USA
		Type of participants	Runners
		Participants' age (years)	MTSS group = 19 ± 1.07
		BMI (kg/m^2^)	NR
		Height (m)	MTSS group = 1.69 ± 0.07
		Duration of symptoms	NR
		Number of participants	18
		Male percentage (%)	50.00%
		Assessment tool (diagnostics)	Clinical examination to assess passive range of motion and muscle strength in the hips and ankles. Plantar pressure analysis was used to quantify mediolateral pressure balance while walking, and 3D motion capture was used to quantify running kinematics.
		Major findings	Runners who developed MTSS demonstrated tighter iliotibial bands, weaker hip abductors, more pressure under the medial aspect of their foot at initial foot contact, foot flat, and heel off, greater contralateral pelvic drop, and greater peak amounts and durations of rear foot eversion during stance phase.
36	Noh et al. (2015) [45]	Design	Case-control study
		Country	Japan
		Type of participants	Soccer players
		Participants' age (years)	MTSS group = 21.4 ± 2.3
		BMI (kg/m^2^)	NR
		Height (m)	MTSS group = 1.75 ± 0.06
		Duration of symptoms	≥ 2 weeks
		Number of participants	10
		Male percentage (%)	100.00%
		Assessment tool (diagnostics)	Fluoroscopic imaging was used to investigate bone movement during landing in running.
		Major findings	The magnitude of angular change for the medial longitudinal arch (MLA) and lateral longitudinal arch (LLA) was significantly greater in subjects with MTSS than in the control subjects. The translational motion of the MLA and LLA in the MTSS group was also significantly greater than that in the non-MTSS group. Soccer players with MTSS have an abnormal structural deformation of the foot during the support (or stance) phase of running, with a large decrease in both the MLA and LLA.
37	Okunuki et al. (2019) [44]	Design	Cross-sectional study
		Country	Japan
		Type of participants	Athletes
		Participants' age (years)	MTSS group = 20.5 ± 1.5
		BMI (kg/m^2^)	MTSS group = 22.0 ± 2.0 kg/m^2^
		Height (m)	MTSS group = 170.7 ± 5.4 cm
		Duration of symptoms	≥ 2 weeks
		Number of participants	22
		Male percentage (%)	90.90%
		Assessment tool (diagnostics)	Segment angles of the hindfoot and forefoot during walking and running barefoot on a treadmill were recorded using three-dimensional kinematic analysis.
		Major findings	Subjects with MTSS exhibited significantly greater hindfoot eversion and abduction during walking and running, forefoot eversion and abduction during walking, and forefoot abduction during running than subjects without MTSS. Participants with MTSS exhibited higher BMI values than those without MTSS.
